# A venom fraction from the Philippine tarantula (*Orphnaecus* sp.) reveals low-molecular-weight compounds that potentiate drug-like neurobehavioral responses in *Danio rerio*

**DOI:** 10.1590/1678-9199-JVATITD-2024-0063

**Published:** 2026-01-30

**Authors:** Joshua Lawrence C. Bautista, Elian Angelo M. Abellanosa, Ralph Emerson John Molino, Jaden G. Jardiolin, Rizelle Anne A. Calpo, Mark Kevin P. Devanadera, Anna Beatriz R. Mayor, Olga M. Nuñeza, Darrell C. Acuña, Camille Rodriguez, Myla R. Santiago-Bautista, Gardee T. Peña, Hiyas A. Junio, Leonardo A. Guevarra

**Affiliations:** 1 Department of Biochemistry, Faculty of Pharmacy, University of Santo Tomas, Manila, Philippines; 2 Secondary Metabolites Profiling Laboratory, Institute of Chemistry, University of the Philippines-Diliman, Quezon City, Philippines.; 3 Research Center for the Natural and Applied Sciences, University of Santo Tomas, Manila, Philippines.; 4 College of Arts and Sciences, Romblon State University, Odiongan, Romblon, Philippines.; 5 Department of Biological Sciences, Iligan Institute of Technology, Mindanao State University, Iligan City, Lanao del Norte, Philippines.; 6 Philippine Arachnological Society Incorporated, Manila, Philippines

**Keywords:** fraction, Low-molecular-weight compounds, Drug-like response, Neurobehavioral response, Spider venom

## Abstract

**Background:**

Spider venoms are rich natural sources of bioactive chemicals ranging from low-molecular-mass compounds to larger molecules such as low molecular mass peptides, proteins, and enzymes. Some compounds have been reported to exhibit neuroactivity and show potential as therapeutic agents against neurological disorders. Thus, this study analyzed the neurobehavioral effects of selected venom fractions from Philippine tarantula species compared to FDA-approved drugs targeting neuroreceptors, ion channels, and enzymes.

**Methods:**

The venom was collected from the tarantula by electrostimulation and fractionated by reverse-phase high-performance liquid chromatography (RP-HPLC). Nine of the eleven fractions were subjected to neurobehavioral analysis using zebrafish (*Danio rerio*) as the animal model. The fractions were administered intraperitoneally, and their neurobehavioral effects were examined using the novel tank test, fear response, social interaction, and mirror biting tests. Donepezil, lidocaine, and diazepam were used as positive controls, and normal saline solution (NSS) as the negative control of the study. The swimming patterns and trajectories of the zebrafish were monitored using idTracker and were graphed using GraphPad Prism v.9.0. Components of the most polar fraction were further analyzed by Ultra Performance Liquid Chromatography - Quadrupole Time of Flight Data Dependent Analysis to identify the components structurally.

**Results:**

Preliminary screening of all the fractions revealed that Fraction 1 with 0.1 µg/µL exhibited donepezil-like behavior based on similar rapid-swimming movement from 0 to 31 time intervals, Fraction 4 with 0.1 µg/µL concentration exhibited diazepam-like behavior due to non-significant differences in its time spent on top of the tank ranging from20 to 40 minutes, and Fraction 8 with 0.1 µg/µL concentration exhibited lidocaine-like behavior based on both rapid swimming movement and time spent on top of the tank. Fractions 1, 4, and 8 were further evaluated by determining their dose-dependent response, which follows the effect of their corresponding positive control. Analysis of Fraction 1 resulted in the annotation of several non-peptidic components 4-OH-PhLac434 and its isomer using VenoMS and isopimaric acid, palmitamide, 9-octadecenamide, and 13-docosenamide as putative compounds present in this spider venom using GNPS.

**Conclusion:**

Overall, the fractions of venom from the *Orphnaecus* tarantula species appear to induce distinct neurobehavioral effects, which may include hyperactivity, anxiolytic-like responses, and potential antinociceptive properties.

## Background

Venom production is an evolutionary adaptation that serves both offensive and defensive roles-enabling prey capture, digestion, and protection from predators [1]. Animal venoms are complex mixtures of bioactive molecules that act on several molecular targets, including ion channels, receptors, and enzymes [2, 3]. Many of these compounds, particularly those from arthropod venoms, are neuroactive and can modulate both the central and peripheral nervous systems [4].

Arthropod venoms are of special interest because of their chemical diversity. They are rich in peptide inhibitors that modulate neuronal activity by blocking or altering potassium channel gating [5, 6]. Neurotoxins from scorpions, snakes, and bees have shown potential as treatments for inflammatory and neurodegenerative diseases-not only for their effects on neural signaling but also for their antimicrobial properties [7, 8]. Spider venoms contain four main classes of compounds: low-molecular-mass compounds (LMMCs), antimicrobial peptides, neurotoxic peptides, and various proteins and enzymes [9]. Among these, LMMCs and peptide neurotoxins are particularly promising for neurological applications, as they modulate ion channels and receptors in excitable neurons. This has been demonstrated in species such as *Cupiennius salei* and *Aphonipelma hentzi* [10].

The Philippines, known for its unique ecosystem and vast biodiversity, is also home to diverse tarantula species. This abundance of spiders has paved the way for research on the neurologic bioactivities of spider venoms. The venom of *P. bundokalbo* contains components that induce apoptosis and necrosis in human lung adenocarcinoma (A549) cells, as well as promoting the proliferation of human breast cancer cells (MCF7) [11, 12]. Although studies on Philippine tarantula venoms have primarily focused on their potential as chemotherapeutic agents against tumor cells, these specific toxins may also possess neurotoxic properties-highlighting the complex and multifaceted roles of tarantula venom components. The foundational evidence indicates the potential of Philippine spider venom. There are only a few journals that have focused on the possible applications of the bioactive components found in these spider venoms. When analyzed, the spider venom fractions have been elaborated more on their cytotoxicity rather than neuroactivity [13, 14, 15]. Thus, more extensive research about the neuromodulatory capacity of selected Spider venom fractions would enable its advanced application in the field of neurosciences.

This study addresses this gap by examining venom fractions from the Philippine *Orphnaecus* tarantula species. It aims to identify which fractions exhibit neuroactivity and to assess their effects on zebrafish behavior, whereas explores the possible use of these components as neuromodulatory agents for neurological conditions.

Previous research has demonstrated that certain spider venom fractions, such as RT10 from *Parawixia bistriata*, can protect neurons from excitotoxic damage in cell cultures [16]. In addition, zebrafish models have also been used to study spider toxins that affect mammalian ion channels, highlighting their value for behavioral and pharmacological screening [15]. Due to its well-characterized nervous system and genetic similarity to humans, the zebrafish (*Danio rerio*) has become a widely adopted animal model for studying neurological function, dysfunction, and modulation [17]. Zebrafish share a high degree of neurological and behavioral resemblance with humans, particularly in neurochemical pathways and behavioral patterns [18, 19]. These similarities make zebrafish an ideal model for investigating neurobiology in the context of human diseases and for screening potential therapeutic agents. Furthermore, their transparent embryos, rapid development, and ability to perform high-throughput screening allow for detailed observation of brain development, neuronal activity, and behavioral responses to drugs, offering valuable insights into neurospecificity and drug efficacy.

This work provides a platform for identifying novel bioactive compounds with potential relevance to human neurological diseases by integrating venom fractionation and zebrafish behavioral assays.

## Methods

### Spider collection and identification

Spiders were collected from Barangay Amoyong in Wao, Lanao del Sur, Philippines (Wildlife Gratuitous Permit R10-2017-35). Spiders were identified by morphological analysis of embolus, chelicerae, maxilla lyra, and palpal patella. Ethical review and approval on the use of spiders were not required for the study on invertebrate animals in accordance with the local legislation and institutional requirements as reviewed by the University of Santo Tomas - Institutional Animal Care & Use Committee (UST-IACUC). A specialist is necessary for the identification of spiders collected from a single location. Notably, the qualitative composition of the venom.

### Venom extraction and fractionation

Venom was extracted via electrical stimulation after halting feeding for 7 days [20]. The collected spiders were placed in a plastic container, followed by anesthetization using carbon dioxide gas (CO_2_) for 5 to 10 min. Anesthetized spiders were retrieved, and their fangs were washed with distilled water for 1 min and positioned in a 1.5 mL tube to collect venom discharges from the fang tip. Stimulator electrodes were placed in contact with the fissure between the cephalothorax and chelicerae. The voltage setting for stimulation was 12-V electricity. The extracted samples were subjected to centrifugation, lyophilization, and storage at -20 °C. Venom extractions were performed at 2-week intervals to allow the tarantula to replenish its venom supply.

The collected crude venom was fractionated by reverse-phase high-performance liquid chromatography (RP-HPLC) following the protocols by Lopez et al. [14] and Santiago-Bautista et al. [21]. The collected samples from three consecutive extractions were pooled and diluted with 100 μL of a 1:1 ratio of 1% TFA in water (Solvent A) and 1% TFA in 90% acetonitrile (Solvent B). Fractionation was performed using a Waters e2695 reverse-phase high-performance liquid chromatography (RP-HPLC) system and an Agilent Eclipse Plus C18 column (5 um, 4.6 x 150 mm). One hundred microliters of diluted venom was injected. Separation was performed in a linear gradient of 5% to 65% of Solvent B over 90 min. The elution of the venom components was monitored at 215 nm, and fractions were collected according to the appearance of peaks [22]. Fractions collected were immediately lyophilized and stored at -20 ^o^C. The use and storage of spider venom and its fractions in all experiments are reviewed and approved by the Institutional Biosafety Committee of the University of Santo Tomas (UST-IBC) with expectations that the experiments should be carried out with maximum safety considerations.

### Maintenance and housing conditions of zebrafish

Wild-type zebrafish strains were obtained and maintained under standard conditions according to the protocols established by Aleström et al. [23]. Zebrafish were housed in a 20-liter recirculating tank system equipped with a contaminant filter, germicidal UV, and dechlorinating solution. The water quality was maintained at a temperature range of 24 °C to 29 °C. A standardized natural photoperiod of 14-hour light/10-hour dark was utilized. Zebrafish were fed dry feed (brine shrimp) two to three times daily in pinch amounts. Size and weight were controlled factors; only adult zebrafish of relatively similar size (3-5 cm in length; 0.7-1.2 cm in width) and an average weight of approximately 3 g were selected for behavioral assays. All experimental procedures involving the use of zebrafish were reviewed and approved by the Institutional Animal Care & Use Committee of the University of Santo Tomas (UST-IACUC) with a review protocol code of RC2018-760708.

### Preparation and administration of venom

The lyophilized venom fraction was reconstituted in phosphate-buffered saline (PBS) and diluted to three concentrations: 0.1, 0.15, and 0.2 µg/µL to evaluate dose-dependent effects in zebrafish. Before injection, zebrafish were individually weighed and anesthetized in ice water (0 °C-4 °C) for approximately 2 min. The venom was administered via intraperitoneal injection following a modified protocol from Kinkel et al. [24]. Five microliters of the venom solution sample were injected using either a 34-gauge 25 μL Hamilton syringe or a 10-μL precision syringe. Each anesthetized zebrafish was positioned on a slitted surgical sponge during injection to ensure proper restraint and targeting.

### Neurobehavioral tests

*Preliminary screening*

Nine fractions were used for preliminary screening. Three substances were tested as behavioral references, namely, donepezil, lidocaine, and diazepam while normal saline solution (NSS) was used as the negative control. Each fish from the control or treatment groups was injected intraperitionally with 0.2 µg/µL of positive control reagent or venom fraction. Behavioral testing commenced immediately after injection and reintroduction to the tank, consistent with the venom-treated groups.

*Novel tank test method*

The experimental zebrafish were allocated to a rectangular tank with dimensions of 30 cm (length), 15 cm (width), and 25 cm (height). During the tank test, the following behavioral endpoints were recorded: average, maximum, and minimum motion speeds; distance traveled; percentage of time spent freezing, swimming, and in rapid movement; time spent at the top of the tank; and the ratio of time spent at the top to that spent at the bottom. Observations began immediately after the fish were injected and reintroduced to the tank. Behavioral responses were recorded for 1 min at time intervals of 0-1, 5-6, 10-11, 15-16, 20-21, 25-26, and 30-31 min. Recordings were conducted daily for 1 week. The video data captured by the camera were analyzed using idTracker software, and the pixel data were converted to centimeters using ImageJ. The trajectory data from idTracker were further processed and calculated in Microsoft Excel, and GraphPad Prism was used to generate the graphs. All video recordings were captured using a Canon DSLR-A550 camera [25].

The behavioral endpoints in zebrafish provide insights into various neurological and psychological phenotypes. The average speed (cm·s⁻¹) serves as an indicator of motor and/or neurological function. Meandering, which corresponds to movement without a fixed direction, is associated with anxiety, with higher values reflecting increased anxiety levels. Freezing time (s) is another anxiety-related measure, in which longer durations suggest heightened stress responses. Although unspecified in interpretation, rapid time movement (s) is typically analyzed in the context of activity or arousal. Time spent at the top of the tank is inversely related to anxiety-higher durations and lower anxiety levels. Thigmotaxis, or the tendency to stay close to the tank walls, is measured by the distance traveled from the center; lower values often imply reduced anxiety. Mirror biting time is used to assess aggression levels because it reflects how frequently a zebrafish engages with its own reflection. Social interaction time evaluates sociability by tracking interactions with other zebrafish. Finally, the predator approaching time assesses a fish’s interaction with a predator stimulus, with lower values indicating higher anxiety levels.

Key behavioral endpoints in zebrafish, such as average speed, freezing time, time spent in the top zone, thigmotaxis, mirror biting, social interaction, and predator approaching time, are widely used to assess neurological, emotional, and social phenotypes [26]. Meandering, which is characterized by irregular movement patterns and associated with anxiety-like behavior, further complements these measures [27]. Rapid movement time supports meandering by indicating erratic locomotion under stress.

*Fear response test*

A transparent glass separator was placed at the center of the tank to isolate the experimental fish from visual stimuli. The convict cichlid (*Amatitlania nigrofasciata*) was used as the predator fish, as it induces fear responses in zebrafish [25]. We followed the same injection protocol and recording setup described in the Novel Tank Test. Each recording lasted for 5 min, and video analysis was performed using idTracker, following the protocol previously described in the novel tank test.

The convict cichlid (*Amatitlania nigrofasciata*) was used as a static visual predator model and positioned approximately 2 cm from the side of the test tank to ensure consistent visibility and perceived threat across trials. The stimulus was introduced following an acclimation period and remained fixed throughout the observation period to minimize experimental variability. The frequency of zebrafish approaches toward predators was recorded as an indicator of fear-related behavior. Increased interaction was interpreted as indicating lower stress and anxiety levels in the zebrafish [26].

*Social interaction test*

A separate glass tank with a transparent divider in the center was used to isolate the experimental fish from its conspecifics. We followed the same injection protocol and recording setup described in the Novel Tank Test. Each session was recorded for 5 min, and the videos were analyzed using idTracker, following the same protocol outlined in the novel tank test.

*Mirror biting test*

Fish injected with a designated venom fraction were placed in a 9.5 L tank and observed immediately after being returned to the water. A mirror was carefully positioned inside the tank to avoid disturbing the fish. Each trial was recorded for 5 min, following the setup used in previous tank tests [28]. Behavioral endpoints were established to interpret the responses of the fish to its own reflection [29].

### Identification of the putative structures of venom fraction composition by UPLC-QTOF

Waters ACQUITY UPLC^®^ I-Class System equipped with an ACQUITY UPLC^®^ CSH™ Fluoro-Phenyl column (1.7 µm 2.1 mm x 50 mm) interfaced with Waters Xevo^®^ G2-XS Quadrupole Time-of-Flight (QToF) mass spectrometer was used to conduct full scan (MS1) and fragmentation studies (MS2) of the fractions. Samples were resuspended in 50% (v/v) acetonitrile (Merck LiChrosolv^®^, Burlington, Massachusetts, USA) to a final concentration of 1.0 mg/mL, vortex and sonicated to completely mix the solution, and filtered using a 0.45 µm syringe filter prior to injection of 3.0 µL sample volume. Chromatography was carried out using LC-MS grade water with 0.1% formic acid (solvent A) and LC-MS grade acetonitrile with 0.1% formic acid (solvent B). Solvent gradient for elution is as follows: 95% A (0.00-0.75 min); 95 to 75% A (0.75-1.00 min); 75 to 50% A (1.00-2.00 min); 50% A to 100% B (2.00-9.00 min); 100% B to 95% A (9.00-9.50 min), and 95% A (9.50-12.00 min) at a constant flow rate of 0.350 mL/min.

Acquisition of full scan MS in the positive ionization mode used the following instrument parameters: capillary voltage at 3 kV; sampling cone voltage at 40 V; source temperature at 150°C; source offset at 80 V; desolvation temperature at 500°C; cone gas flow at 50 L/hr, and desolvation gas flow at 50 L/hr. MS1 scans were acquired at *m/z* 200 to 1000 Da and a scan time of 0.50 seconds. The MS2 analysis was performed through Data-Dependent Acquisition (DDA), in which the instrument alternates between MS1 detection to identify highly abundant ions, which are subjected to MS2 analysis. MS1 scanning was performed on the same mass range and scan time, defining the eight most abundant ions with signal intensities above 3.0x10^5^. MS2 spectra of precursor ions were acquired for 0.5 s, scanning for fragment ions in the *m/z* 50 to 1000 range. The selected ions were accelerated and collided with an argon curtain to promote gas-phase fragmentation. Different profiling runs were performed at manually specified collision energies (15 V, 30-45 V, 45-60 V, and 60-75 V) to obtain comprehensive information on the fragmentation patterns of the metabolites and to enhance spectral library matching in GNPS.

The MSConvert tool of ProteoWizard [30] was used to convert .RAW Waters DDA data to open-source 32-bit .mzXML format. The dataset was uploaded on GNPS [31], which enabled the comparison of sample data to a publicly curated spectral library of reference compounds. The parameters for library matching include precursor ion mass tolerance of 0.02 Da, fragment ion mass tolerance of 0.05 Da, and minimum similarity (cosine) score of 0.70. At least seven matched peaks between sample and reference spectra were needed for an alignment to be considered a spectral match. In addition, mass spectra of highly intense features were compared and matched in the online database VenoMS (University of Zurich, https://www.venoms.ch/, Accessed: November 2022) containing experimental tandem-MS spectra of published small molecule toxins from arachnids.

### Statistical analysis

A total of 3 zebrafish (n = 3) were used in each neurobehavioral test, and all data expressed as mean ± standard error of the mean (SEM). A non-parametric test was used, specifically, the Kruskal-Wallis test, followed by Dunn’s multiple comparison tests in every neurobehavioral data. All statistical analyses were performed using the GraphPad Prism software. Figures depicting the swimming trajectories are representative of multiple independent replicates.

## Results

### Venom fraction

**Figure 1.**
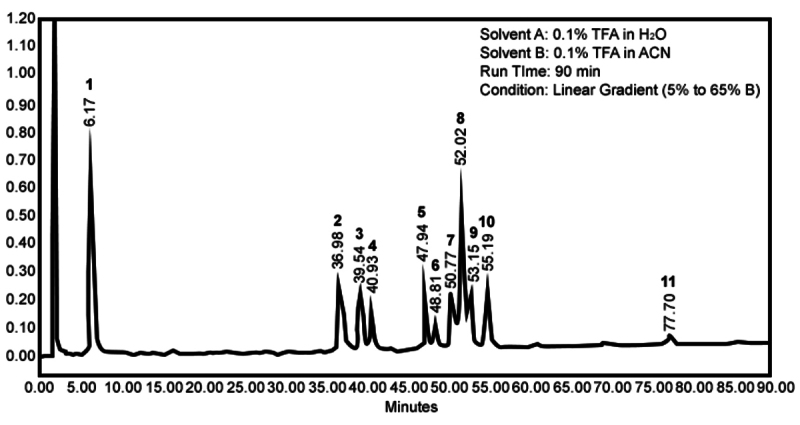
RP-HPLC chromatogram of spider venom. The linear gradient elution of 5% to 65% of solvent B over 90 min resolved the venom into eleven peaks which were collected as individual fractions. It had 11 distinct peaks divided into three groups: polar (Fraction 1; t_R_ = 6.17 min), mid-polar (Fraction 2 with t_R_ = 35.98 min; Fraction 3 with t_R_ = 39.54 min; Fraction 4 with t_R_ = 40.93 min; Fraction 5 with t_R_ = 47.49 min; Fraction 6 with t_R_ = 48.81 min; Fraction 7 with t_R_ = 50.77 min; Fraction 8 with t_R_ = 52.02 min; Fraction 9 with t_R_ = 53.15 min; Fraction 10 with t_R_ = 55.19 min), and least polar (Fraction 11 with t_R_ = 77.70 min).

Linear gradient venom elution produced 11 peaks clustered into three groups based on elution time: polar, mid-polar, and least polar. The peak with a retention time of 6.17 min was considered the polar group (Figure 1). Peaks that eluted between 36.98 min to 55.19 min were regarded as the mid-polar group, were regarded as the mid-polar group (Figure 1). The small peak with a retention time of 77.70 min was considered the least polar (Figure 1).

### Screening of the neurobehavioral activity of spider venom fractions

**Figure 2.**
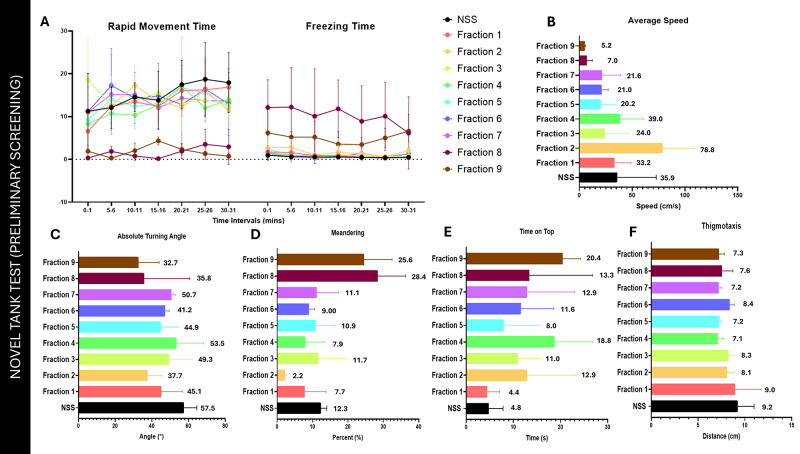
Preliminary Screening of all nine venom fractions observed on the varying behavioral endpoints: **(A)** rapid movement and freezing time, **(B)** average speed, **(C)** absolute turning angle, **(D)** percent meandering, **(E)** time spent on top, and **(F)** thigmotaxis.

Preliminary screening of all venom fractions in Figure 2 showed that Fractions 8 and 9 exhibited a hypoactive effect in zebrafish behavior. It is evident from the rapid and freezing time graph that Fractions 8 and 9 significantly deviate from the negative control. Also, Fractions 8 and 9 obtained the lowest average speed and the highest percent meandering compared to the other venom fractions.

Fraction 1 of the spider venom was also analyzed for subsequent neurobehavioral and dose-dependent assessment (Figure 2). Other venom fractions that exhibited NSS-like behavior for the rapid and freezing time were analyzed further using the other NTT endpoints, such as time spent on top, thigmotaxis, and absolute turning angle. Fraction 4 differed from the other fractions based on the time spent on top (Figure 2). Due to resource constraints involving: the limited amount of venom, unrecorded mass of the 10th fraction, and scarce amount for Fraction 11, the screening only involved Fractions 1 to 9.

### Behavioral swimming response of zebrafish against Fraction 1

Donepezil, an acetylcholinesterase inhibitor, promotes the continued action of the neurotransmitter AChE, towards excitatory neuronal nerve impulses and subsequent hyperactive physiological state via acetylcholine build-up in the neuronal synapses [32]. This is observed for donepezil-injected fish on hyperactive manifestations like increased rapid movement, decreased freezing time, increased average speed, longer time spent on top (about the hyperactivity-induced elevated oxygen requirement), and a lowered percent meandering in terms of the swimming of the zebrafish [33].

*Novel tank test method*

**Figure 3.**
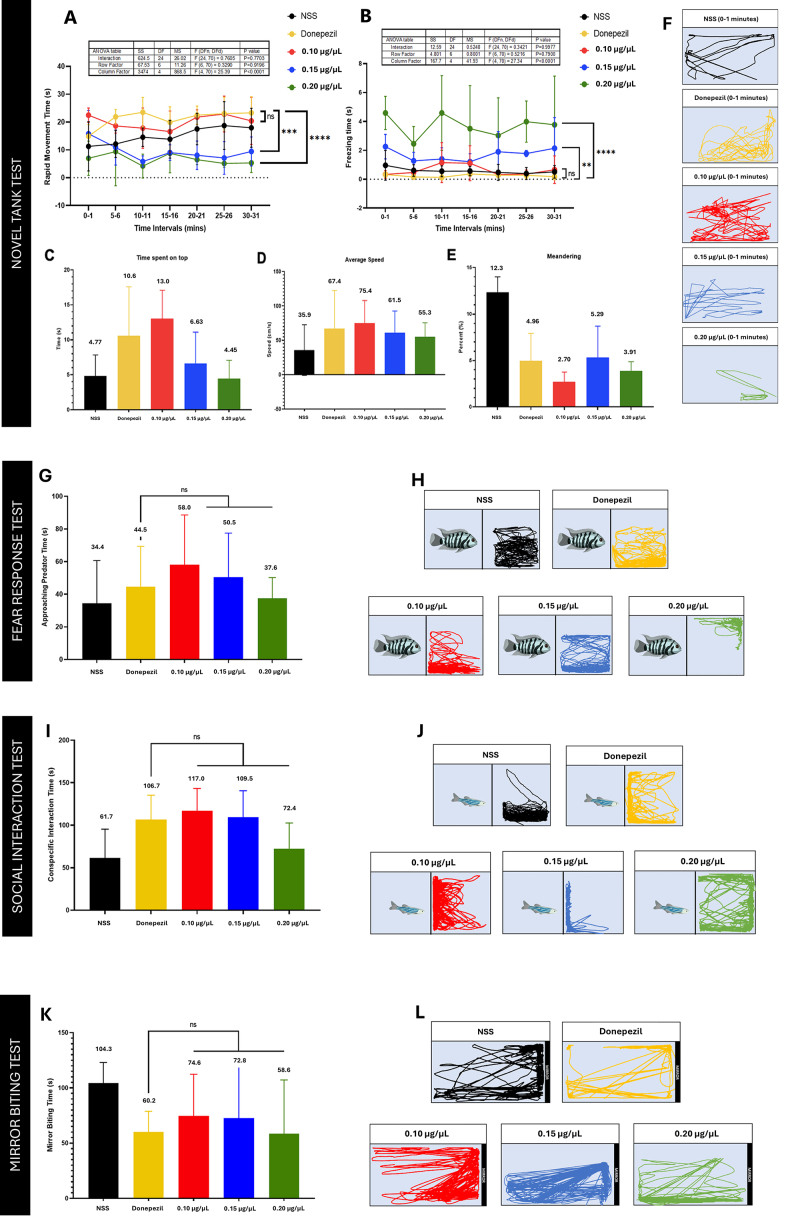
Neurobehavioral response of Fraction 1 in the novel tank test. **(A)** Rapid movement time, **(B)** freezing time, **(C)** average speed, **(D)** time spent on top, **(E)** percent meandering, and **(F)** trajectories at 0-1 min, **(G)** dose-dependent approaching predator time of Fraction 1. Swimming trajectories of **(H)** the sample groups: NSS, donepezil, 0.1 μg/μL, 0.15 μg/μL, and 0.2 μg/μL, **(I)** dose-dependent conspecific interaction time of Fraction 1. Swimming trajectories of **(J)** the sample groups: NSS, donepezil, 0.1 μg/μL, 0.15 μg/μL, and 0.2 μg/μL, **(K)** dose-dependent mirror biting time of Fraction 1. Swimming trajectories of **(L)** the sample groups: NSS, diazepam, 0.1 μg/μL, 0.15 μg/μL, and 0.2 μg/μL.

The NTT results (Figure 3), zebrafish injected with 0.1 µg/µL of Fraction 1 exhibited swimming activity similar to those treated with donepezil. However, an inverse dose-dependent trend was observed at higher concentrations. Specifically, zebrafish treated with 0.15 and 0.2 µg/µL of Fraction 1 showed behavioral patterns that increasingly deviated from those of the donepezil-treated group across all five NTT endpoints-except for percent meandering, which remained comparable.

*Fear response test*

For the fear response test (Figure 3 G-H ), data presented all three concentrations of Fraction 1 to mirror the behavioral effect of donepezil on zebrafish when faced with a pertinent predatory species. The use of 0.1 µg/µL of Fraction 1 led to zebrafish displaying the highest approaching predator time. Similar to the earlier observed trend for NTT, the higher concentrations of 0.15 µg/µL and 0.2 µg/µL resulted in a decreasing time in showing predator-directed response by the tested zebrafish, despite still being considered donepezil-like (p > 0.05).

*Social interaction test*

Sociability results (Figure 3 I-3J ) entailed higher conspecific interaction by the donepezil- and fraction-injected zebrafish relative to the negative control group. Swimming trajectories depict increased proximal swimming of the tested zebrafish to the zebrafish at the other side of the tank for each trial. This suggests that both donepezil and Fraction 1 positively affect zebrafish sociability via intraspecific interaction (p > 0.05).

*Mirror biting test*

Self-aggression (Figure 3 K-3L ) was monitored through zebrafish response time (proximal swimming) to its reflection. Both donepezil- and fraction-injected zebrafish exhibited decreased self-aggression relative to activity displayed by negative control (p > 0.05).

### Behavioral swimming response of zebrafish against Fraction 4

*Novel tank test method*

**Figure 4.**
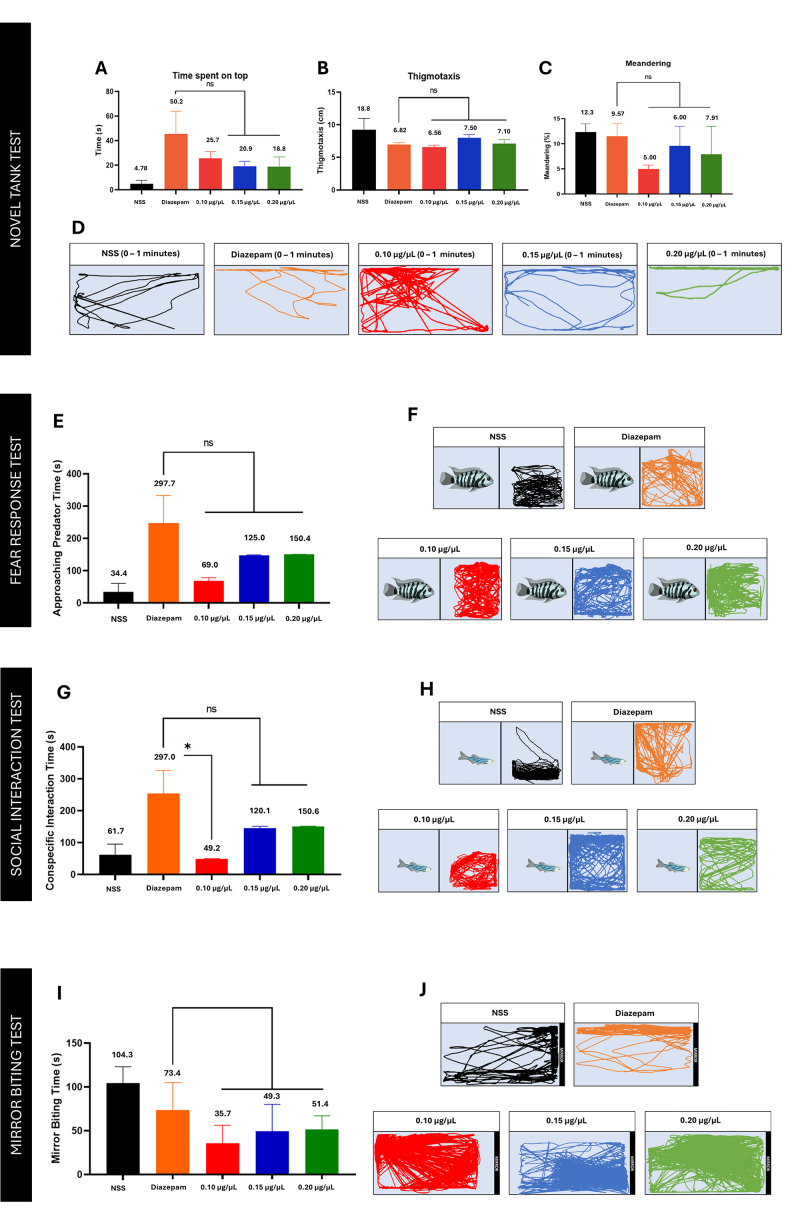
Neurobehavioral response of Fraction 4 in the novel tank test. **(A)** Time spent on top, **(B)** thigmotaxis, **(C)** percent meandering, and **(D)** trajectories at 0-1-min, **(E)** dose-dependent approaching predator time of Fraction 4. Swimming trajectories of **(F)** the sample group: NSS, diazepam, 0.1 μg/μL, 0.15 μg/μL, and 0.2 μg/μL, **(G)** dose-dependent conspecific interaction time of Fraction 4. Swimming trajectories of **(H)** the sample groups: NSS, diazepam, 0.1 μg/μL, 0.15 μg/μL, and 0.2 μg/μL, **(I)** dose-dependent mirror biting time of Fraction 4. Swimming trajectories of **(J)** the sample groups: NSS, diazepam, 0.1 μg/μL, 0.15 μg/μL, and 0.2 μg/μL.

From the novel tank test (Figures 4 A-4D ), diazepam obtained the highest time spent on top, 45.58 s, indicating its potential anxiolytic activity. Zebrafish often exhibit a bottom-dwelling swimming pattern when exposed to stress or an unfamiliar environment. Thus, as the zebrafish spends more time in the upper portion of the tank, the zebrafish becomes less stressed or anxious. A previous study reported a significant reduction in the bottom-dwelling of zebrafish treated with diazepam [34]. The dose-effect function of diazepam was biphasic, which was also observed in the results. Moreover, Fraction 4 of the spider venom exhibited swimming patterns consistent with those of the diazepam-treated zebrafish. In addition, an inverse dose-dependent effect of the fraction was observed from time spent on top, which delineates that as the concentration of the venom fraction increases, the time spent on top decreases. This explains the possible deviations of diazepam as an anxiolytic and sedative, in which high concentrations of diazepam manifest sedative effects in adult zebrafish [35].

All concentrations of the venom fraction showed a distinct increase in their time spent on top, which differs significantly from the negative control (p < 0.05) Other endpoints, such as thigmotaxis and percent meandering, also showed that the venom fraction has no statistical difference in the effect of diazepam in zebrafish (p > 0.05). In addition, diazepam obtained the highest approaching predator time of 247.26 s, which shows how it acts as an anxiolytic by lowering the fear and anxiety of zebrafish and other previously reported animal models [36, 37].

*Fear response test*

The fear response test revealed a distinct increase in the approaching predator time of both diazepam and venom fraction. These results further supplemented the anxiolytic properties of the venom fraction as it obtained no statistical difference in the behavior exhibited by diazepam-treated zebrafish (p > 0.05) (Figures 4 E-4F ). Likewise, all venom concentrations increased zebrafish’s approaching predator time, showing how the fraction can modulate fear-potentiated response in zebrafish. The venom fraction also exhibited a dose-dependent neurobehavioral effect having 0.2 µg/µL as the concentration that obtained the highest approaching predator time of 150.44 s.

*Social interaction test*

Figure 4 G-H  shows that the sociability of zebrafish treated with diazepam and venom fraction significantly differed from the negative control’s sociability. Diazepam obtained the highest conspecific interaction time of 254.16 s, depicting its anxiolytic effect as it increases the sociability of the experimental fish towards its conspecific. On the other hand, 0.1 µg/µL of the venom fraction did not exhibit the same behavior as the diazepam-treated zebrafish. However, a dose-dependent response was observed in which the zebrafish exhibited a diazepam-like treated behavior as the concentration of the venom fraction increased (p < 0.05).

*Mirror biting test*

Diazepam and the venom fraction significantly decreased zebrafish aggression, with a mirror biting time of 73.44 s. Notably, among the tested venom concentrations, the 0.1 µg/µL dose exhibited the greatest reduction in biting behavior, suggesting that this concentration may be the most effective in modulating aggression-related responses. Overall, the neurobehavioral effects of Fraction 4 of the spider venom exhibited the same anxiolytic effects as those of diazepam (p > 0.05).

### Behavioral swimming response of zebrafish against Fraction 8

Screening the two mid-polar fractions, Fraction 9 failed to exhibit dose-dependent responses for NTT endpoints. Fraction 8 showed dose-dependent responses resembling the behavioral activity shown by lidocaine.

*Novel tank test method*

**Figure 5.**
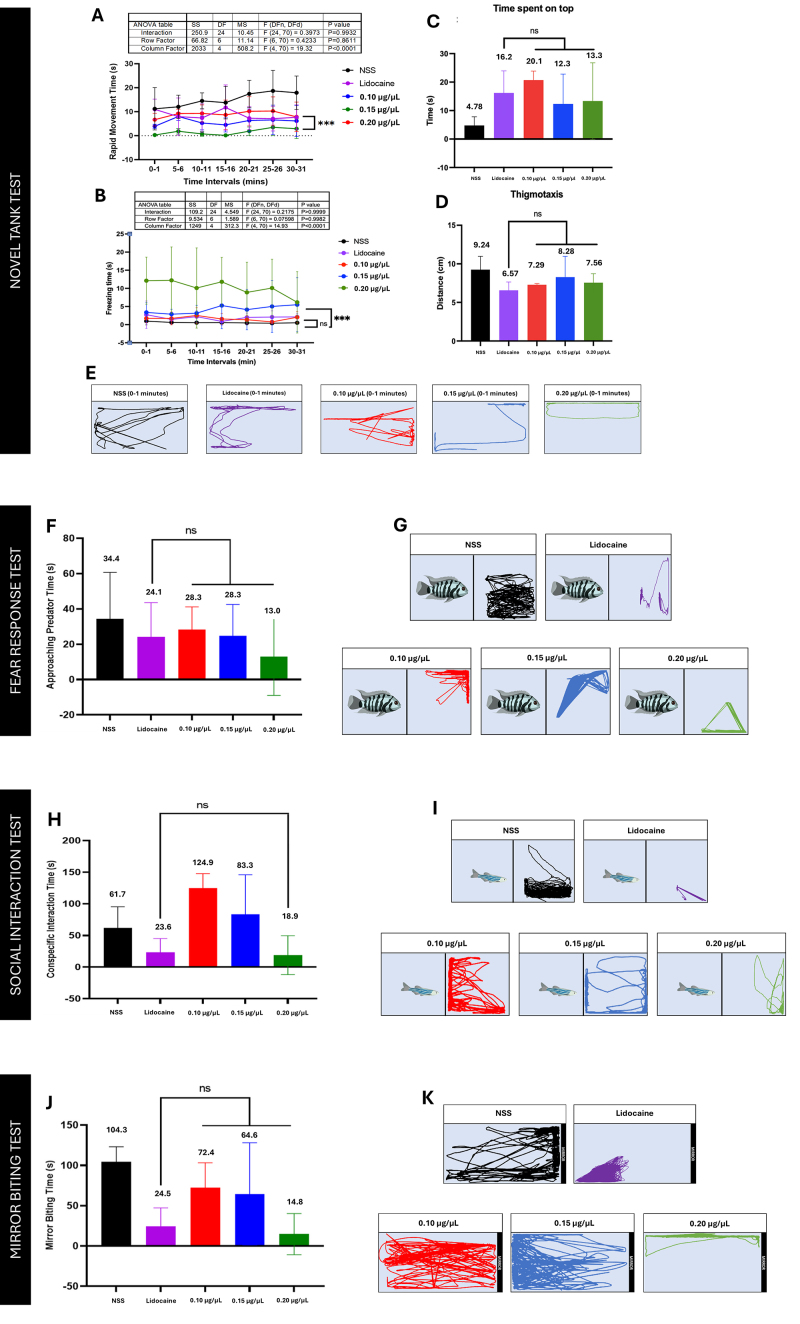
Neurobehavioral response of Fraction 8 in the novel tank test. **(A)** Rapid movement time, **(B)** freezing time, **(C)** time spent on top, **(D)** thigmotaxis, and **(E)** trajectories at 0-1 min, **(F)** inverse dose-dependent approaching predator time of Fraction 8. Swimming trajectories of **(G)** the sample groups: NSS, lidocaine, 0.1 μg/μL, 0.15 μg/μL, and 0.2 μg/μL, **(H)** inverse dose-dependent conspecific interaction time of Fraction 8. Swimming trajectories of **(I)** the sample groups: NSS, lidocaine, 0.1 μg/μL, 0.15 μg/μL, and 0.2 μg/μL, **(J)** inverse dose-dependent mirror biting time of Fraction 8. Swimming trajectories of **(K)** the sample groups: NSS, lidocaine, 0.1 μg/μL, 0.15 μg/μL, and 0.2 μg/μL.

Based on the NTT endpoints, treatment of 0.1 µg/µL concentration showed an identical behavior as with lidocaine-treated groups. At 0.2 µg/µL concentration of the fraction, it showed lower rapid movement compared to lidocaine-treated fish (***p < 0.0001) (Figures 5 A-5E ). The highest concentration exhibited the most time spent frozen in freezing time.

*Fear response test*

The presence of fear stimuli decreased the response to approaching predator time as fraction concentration increased (p > 0.05). For 0.1 µg/µL, it showed an increased value for approaching predator time, indicating lower anxiety [20]. As for 0.2 µg/µL, zebrafish treated with the concentration exhibited behavior comparable to lidocaine, indicating heightened anxiety. The activity visualized (Figures 5 F-5G ) anxious zebrafish swimming away from the stimulus, a common response of the animal model in the presence of a threat [21, 38].

*Social interaction test*

In assessing social interaction behavior, a decrease in conspecific interaction time with a paired zebrafish was observed as the fraction concentration increases. However, with respect to the drug control lidocaine, 0.2 µg/µL is the only concentration that showed no statistical difference (p > 0.05); observably, the zebrafish retained at the far left of the tank, away from the other fish (Figures 5 H-5I ). Notably, the treatments at 0.1 µg/µL and 0.15 µg/µL demonstrated increased social interaction compared with higher concentrations, highlighting the relevance of these concentrations in promoting social behavior. This finding is significant as it suggests that lower-to-moderate venom concentrations may have a positive effect on sociability.

*Mirror biting test*

Testing aggression among the controls using the Mirror biting test, there is no statistically significant difference among the fraction concentrations relative to results for lidocaine (p > 0.05). The 0.2 µg/µL -treated zebrafish responded less to its reflection (Figures 5 J-5K) than lower concentrations.

### Untargeted metabolomics analysis of the spider venom fraction

**Figure 6.**
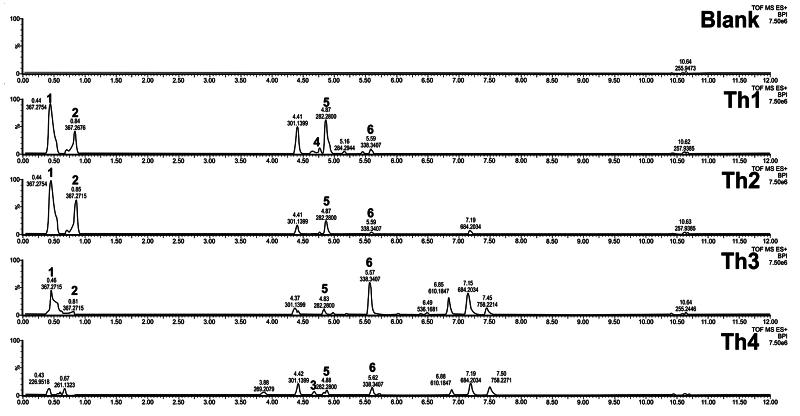
Base peak chromatogram of spider venom extracts. Profile shown corresponds to the resuspension blank (Blank) and fraction of interest from four spider samples collected from the site (Th1 to Th4). Metabolites putatively identified are polyamine isomers of 4-OH-PhLac343 (1, 2), isopimaric acid (3), palmitamide (4), 9-octadecenamide (5) and 13-docosenamide (6). the acylpolyamine 4-OH-PhLac343 (1, 2), isopimaric acid (3), palmitamide (4), 9-octadecenamide (5) and 13-docosenamide (6).

Among the three active fractions, only Fraction 1 was selected for untargeted metabolomics due to volume limitations of Fraction 4 and Fraction 8. The constituents in the venom fraction eliciting hyperactive behavior were profiled using Waters UPLC-QTOF. Figure 6 shows the UPLC chromatogram and GNPS and VenoMS database matches. Close similarity in the full-scan chromatograms of the venom fraction of interest was observed among the four spiders in terms of unretained polyamines (t_R_= 0.40 to 0.80 min) and fatty acid molecules (t_R_ = 4.25-5.50 mins). The 9-octadecenamide (t_R_ = 4.83 - 4.88 min) and 13-docosenamide (t_R_ = 5.57 - 5.62 min) were present in all the samples. Three of the four samples (Th1-Th3) contain 4-OH-PhLac343 (t_R_ = 0.44-0.46 min) and its isomer (t_R_ = 0.81 - 0.85 min). Only the Th1 sample contained palmitamide (t_R_ = 4.71 min) while only the sample from Th4 has isopimaric acid. Further details on the putative identification of metabolites through GNPS and VenoMS databases are available.

**Figure 7.**
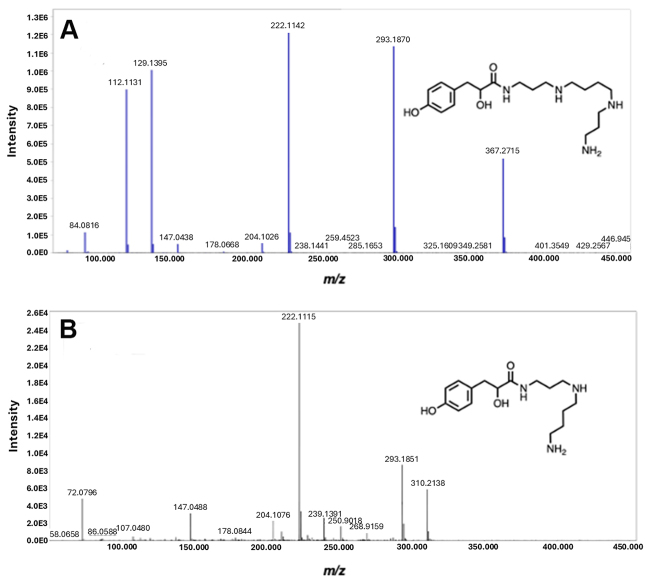
MS/MS spectra of acylpolyamines with *m/z* of 367.2715 and 310.2136. The fragmentation pattern in **(A)** matched with 4-OH-PhLac343 in the VenoMS library. The annotation of the unreported *m/z* 310.2136 in **(B)** is based on similar product ions with *m/z* 367.2715 and unique fragmentation products that further supported its annotation as the shorter, 4-OH-PhLac34.

The MS2 spectra of suspected isomeric compounds with *m/z* 367.2157 (Figure 7 A ) were not matched to a reference file in GNPS. Instead, the VenoMS library singled out the polyamine 4-OH-PhLac343 as the closest hit with respect to the product ions and their intensities. Forster et al. obtained the reference spectra for 4-OH-PhLac343 using a hybrid FT-Orbitrap instrument [39]. Published report by Wilson et al. [22] was cross-referenced for the detailed QTOF spectra of 4-OH-PhLac343, which contains a lipophilic tyrosine head group and a polyamine tail consisting of spermine and spermidine that are linearly connected.

**Figure 8.**
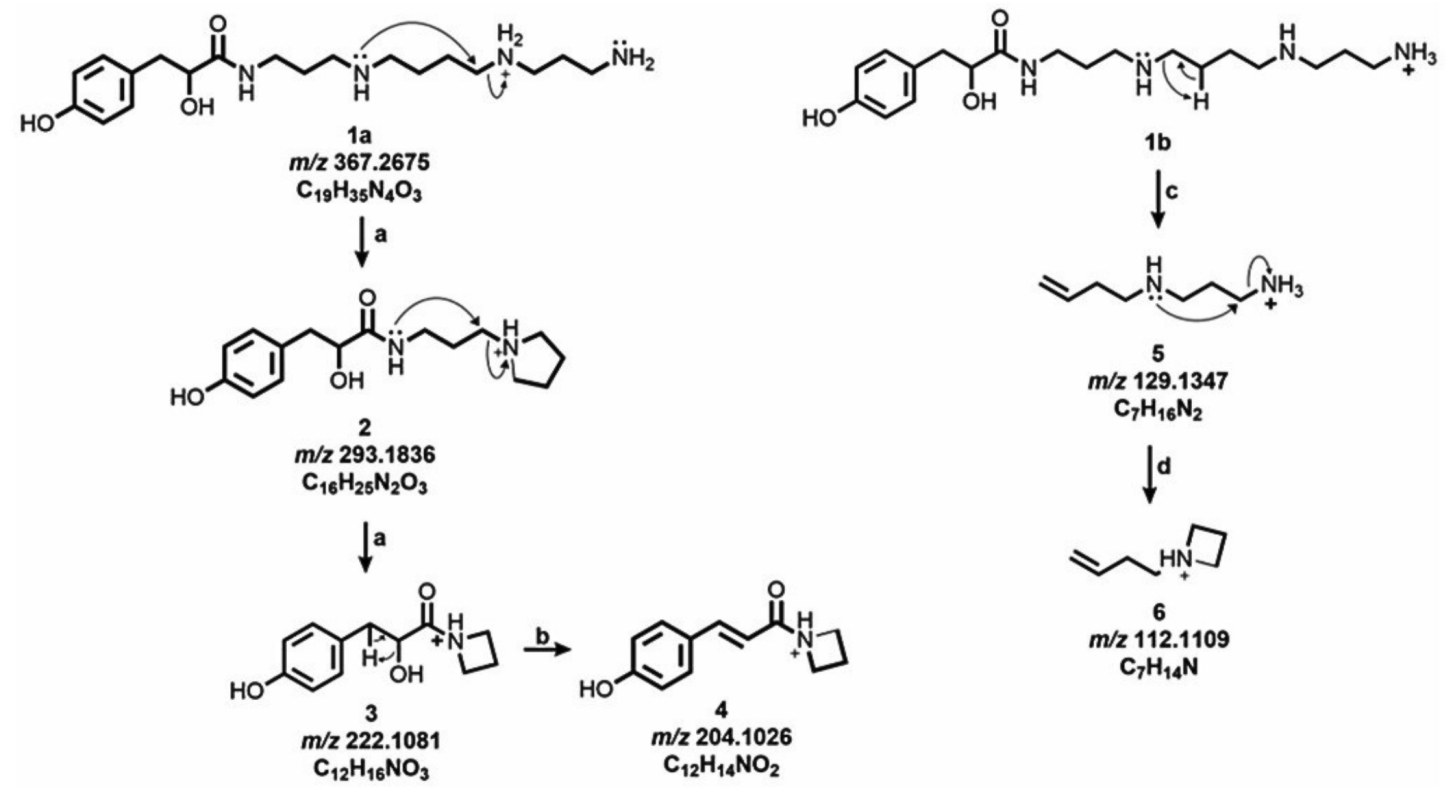
Proposed fragmentation scheme for 4-OH-PhLac343 Theoretical fragmentation of 4-OH-PhLac343 resulted in product ions observed in Figure 5A. Reactions involved are: **(a)** nucleophilic attack, **(b)** loss of H_2_O with charge retention, **(c)** remote H-rearrangement, and **(d)** elimination of NH_3_ with charge migration.

**Figure 9.**
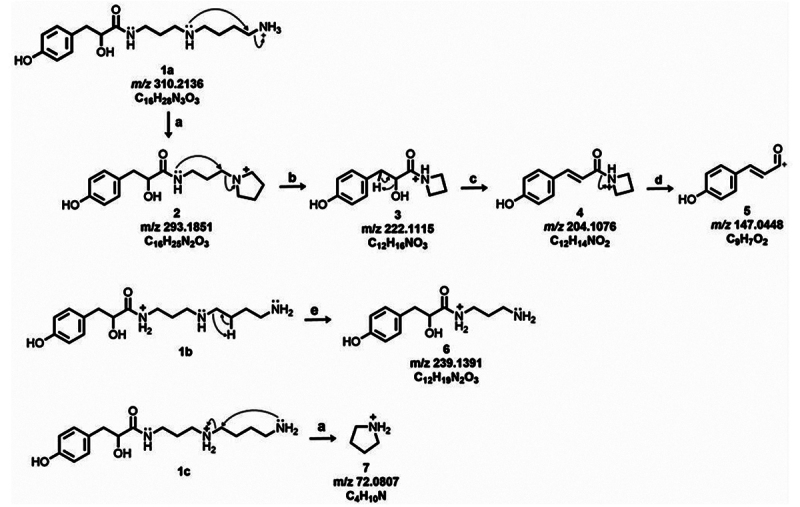
Proposed fragmentation scheme for 4-OH-PhLac34, a shorter analog of 4-OH-PhLac343. Reactions that give rise to product ions include **(a)** loss of NH_3_, **(b)** nucleophilic attack, **(c)** loss of H_2_O, **(d)** inductive cleavage, and **(e)** remote H-rearrangement.

Co-eluting with this major acylpolyamine yielded *m/z* 310.2136, which also yielded *m*/z 293.1851 and *m/z* 222.1115 product ions (Figure 7 B ). Although having lower signal intensity and exhibiting similar product ions with 4-OH-PhLac343, *m/z* 310.2136 was not considered an in-source fragment since it is not a product ion of *m/z* 367.2715. It is immediately hypothesized to be a shorter analog of 4-OH-PhLac343, and the structural elucidation of this unreported acyl polyamine was performed side-by-side with the theoretical fragmentation analysis of 4-OH-PhLa343 (Figures 8 and 9).

The proposed gas-phase reactions of 4-OH-PhLac343 (Figure 8) yielded product ions observed in the experimental mass spectra. Two types of reactions yielded highly intense product ions. The first is a nucleophilic substitution that liberates spermidine and spermine units (Figure 8 reaction a). The other route involves remote H-rearrangement (Figure 8 reaction b), which eliminates the aromatic head group giving rise to low molecular weight product ions with *m/z* 129.1347 and *m/z* 112.1109.

Similarly, the highly intense *m/z* 293.1851 and *m/z* 222.1115 were used to infer the presence of an acylpolyamine head group in the *m/z* 310.2136 precursor ion (Figure 9). The formation of product ions 6 and 7 accounted for the presence of a spermidine and spermine tail.

**Figure 10.**
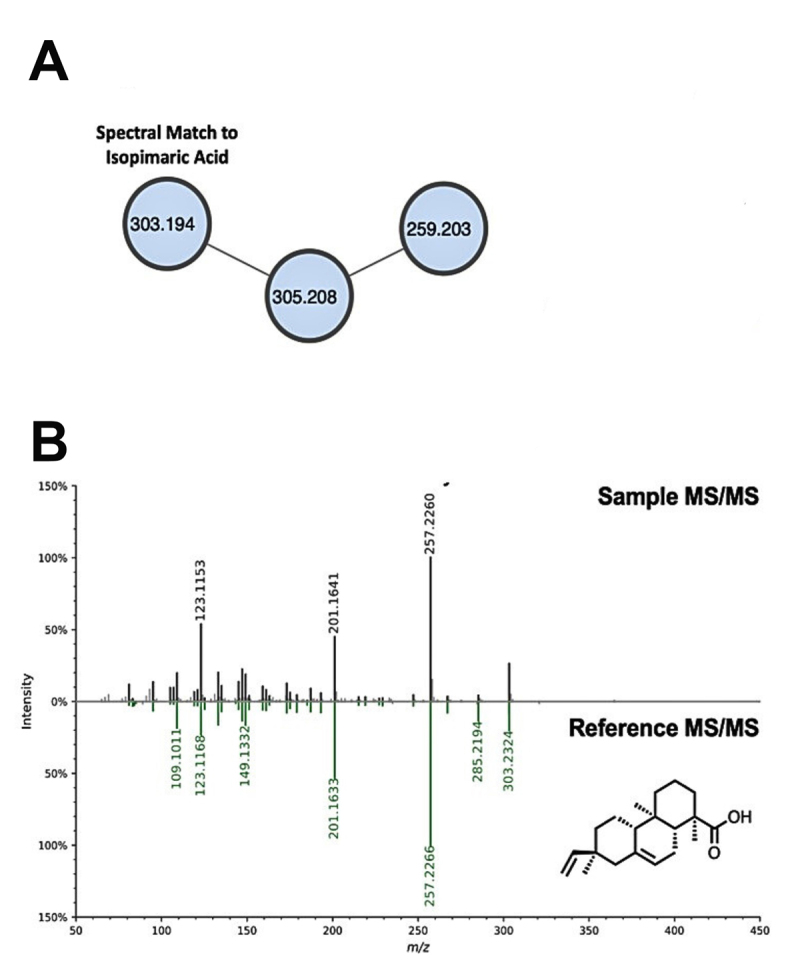
Bioinformatics-based identification of isopimaric acid by GNPS. **(A)** A molecular network with two nodes corresponding to resin acids from the venom fraction. Good alignment between the sample spectra (black) and the reference (green) file in **(B)** the GNPS library, suggests high confidence in the isopimaric acid annotation.

The absence of polyamine toxin in the Th4 sample put emphasis on hydrophobic diterpenoids detected only on the mentioned fraction. Molecular networking aided this annotation (Figure 10 A ). An unidentified precursor ion with *m/z* 305.208 is linked to the [M+H]^+^ ion of isopimaric acid, putatively identified by GNPS Figure 10 B . Tail-to-tail matching affirms strong similarity between sample (black) and reference (spectra) in terms of product ions and their relative abundance. Further examination of the fragmentation pattern provided insights on structural similarities and differences between isopimaric acid and its suspected analog. The presence of the carboxylic acid functional group was associated with losses of H_2_O and CO via charge migration mechanisms. The remaining product ions were attributed to elimination of ethenyl group (-28 Da) and cross-ring cleavage products of the sesquiterpenoid core (below *m/z* 203.18). Furthermore, an approximately 2.02 Da difference between the product ions of the unidentified analogue (*m/z* 259.24, 203.18, 189.16, etc.) and protonated isopimaric ions (*m/z* 257.23, 203.18, 189.16, etc.) hints the absence of a double bond on the backbone of the former. The presence of only sp3 hybridized carbons in this analogue, 1,4a,7-trimethyl-7-vinyltetradecahydrophenanthrene-1-carboxylic give rise to comparable intensities of product ions below *m/z* 203.18.

## Discussion

Spider venom components may comprise compounds with varying levels of polarity based on each component’s chemical nature [40, 41]. The garnered chromatogram likewise reflects highly differing polarities, noting how the three mentioned clusters are of three distinct regions along the elution time axis. The diversity of chemical classes, under which spider toxins constituents fall, contributes to these polarities, ranging from possible nonpolar fatty acid classes [42] to the more polar low-molecular weight compounds like polyamines and biogenic amines [41]. Notably, the venom fractions used in this study, including those identified as behaviorally active (Fractions 1, 4, and 8), were not pure compounds but rather mixtures of co-eluting components. While mass spectrometry allowed us to annotate several putative neuroactive compounds in Fraction 1, such as acyl polyamines, these were detected within complex venom fractions and not as individually purified molecules. Thus, behavioral comparisons between these fractions and standard pharmacologic controls (e.g., lidocaine, diazepam, and donepezil) were intended for qualitative reference only-not as direct potency or purity equivalence.

Based on the behavioral endpoints for the preliminary screening, Fractions 1 and 2 exhibited hyperactive activity. Previous studies reported that the most polar fraction contains a non-peptidic bioactive compound [41]. Given that donepezil is a non-peptidic control drug and Fraction 1 induced similar hyperactive behavior, a qualitative comparison was drawn between their behavioral effects. However, we do not claim mechanistic similarity without further functional assays. Provided that no statistically significant differences were established computed for the 0.1 µg/µL employment results in comparison to donepezil-injected trials, across all five endpoints in the NTT, 0.1 µg/µL of Fraction 1 was regarded as hyperactive or donepezil-like in terms of effect. As for the higher fraction concentrations, 0.15 µg/µL and 0.2 µg/µL, the decrease in their donepezil-like effect was denoted as having a hypoactive effect. This phenomenon may be attributed to the acute toxicity effect of the hyperactive fraction, like the reported toxicity by excessive AChE inhibition in zebrafish [18, 43, 44].

To further contextualize these observations, the known behavioral and pharmacologic effects of donepezil in zebrafish and how they parallel the responses seen with 0.1 µg/µL of Fraction 1. Donepezil is an acetylcholinesterase inhibitor that promotes acetylcholine accumulation in synapses, enhancing excitatory neurotransmission and inducing a hyperactive behavioral state [32]. In our study, zebrafish injected with donepezil showed increased rapid movement, reduced freezing time, elevated average speed, greater time spent on top (linked to increased oxygen demand), and decreased meandering-all consistent with hyperactivity [33].

Moreover, donepezil elevates epinephrine and norepinephrine levels in zebrafish [43], suggesting its effects may extend beyond the cholinergic system. This could explain the increased fear response observed in donepezil-treated fish, and by extension, in those treated with Fraction 1. Additionally, donepezil improves cognitive function and modulate aggression [44-46]. The observed reduced mirror-biting behavior may reflect such cognitive enhancement, with Fraction 1 showing similar behavioral modulation. Increased social interaction in zebrafish has also been linked to cognitive improvement. The similarity between donepezil- and Fraction 1-treated groups in this domain supports the hypothesis that the fraction may share behavioral effects with donepezil. However, the inverse dose-dependent trend-where higher Fraction 1 concentrations result in reduced hyperactivity-may be due to acute toxicity diminishing its primary effect [22, 47].

The selection of the fraction for metabolomic analysis heavily relied on the capability of the remaining fraction volumes for MS/MS analyses. With literature on neurotoxic low-molecular-weight, non-peptidic components of the Philippine spider venom, a typical class eluted in a predominantly polar solvent system in RP-HPLC, hence the founded focus on Fraction 1 [22, 47, 48]. The results of the mass spectrometry analysis confirm the presence of low molecular weight compounds in the fraction of interest which showed significant neurobehavioral activity. The acyl polyamine 4-OH-PhLac343, one of the annotated compounds in Fraction 1, is a neurotoxic compound, first discovered in the 1980s from the orb-weaver spiders belonging to the family Araneidae [49]. Molecules belonging to the same classification with neurotoxic and cytotoxic activity have also been reported in trap-door spiders (*Halonoproctiadae*) and different tarantula species (*Theraphosidae*) [22, 50]. The mode of action of this toxin family involves the disruption of key excitatory glutamatergic transmission [51]. Excessive firing of glutamate neurotransmitters is often implicated in epilepsy as well as neurodegenerative diseases, such as ALS, Huntington’s, Parkinson’s, and Alzheimer’s disease [48].

Another non-peptidic component putatively identified through GNPS is isopimaric acid with *m/z* = 303.2311; t_R_ = 4.26 mins). Molecular networking analysis emphasized the occurrence of its structural analog, the unreported diterpenoid 1,4a,7-trimethyl-7-vinyltetradecahydrophenanthrene-1- carboxylic acid isopimaric acid, a triterpenoid acid, is reported to be an induced chemical defense in *P. pinaster* and *P. radiata* by some chewing insects [52]. The presence of this resin acid in spider venom alludes to the ability of the insect to detoxify and sequester metabolites from plants, and used it as part of its own defense [53]. Sequestration of secondary plant metabolites by insects has been previously investigated [54]. GC-MS, the oral effluent of European pine sawfly (*Neodiprion sertifer*) was found to have high levels of resin acids from scotch pine (*Pinus sylvestris).* The venom profile of the spider can also be related to its feeding habits and habitat.

Moreover, the behavioral effects observed in zebrafish treated with Fractions 4 and 8 may be explained by their similarity to diazepam and lidocaine, respectively, although further biochemical and neurotransmitter analyses are required to confirm these mechanisms. Fraction 4-treated zebrafish spent more time in the top area of the tank, a behavior commonly interpreted as reduced anxiety in zebrafish models [33, 54]. This is consistent with the anxiolytic-like effect of diazepam, a benzodiazepine known to reduce anxiety-related behaviors [55]. Although the specific molecular targets involved cannot be confirmed, the behavioral similarity suggests that Fraction 4 may modulate stress-related pathways in a manner comparable to diazepam.

Meanwhile, zebrafish treated with Fraction 8 exhibited hypoactivity similar to that of the lidocaine control group. Lidocaine, a known voltage-gated sodium channel (VGSC) blocker, exerts antinociceptive effects by inhibiting action potential propagation [56]. The reduced locomotor activity observed in both the lidocaine and Fraction 8 groups may reflect interference with neuromuscular signaling pathways through sodium channel inhibition [57, 58]. The neurobehavioral and neuroactive implications point to its potential as a voltage-gated ion channel inhibitor [59, 60].

The hypoactivity of the lidocaine control group was caused by the inability of the neuromuscular junctions to receive the depolarization product of neurons since muscles require action potential to initiate contraction and relaxation [61]. The hypoactivity of zebrafish can be correlated with the disruption of coordination signaling caused by NaV blockers affecting the cholinergic neurotransmission [54]. As for the time spent on top and thigmotaxis, there are no statistically significant differences that were observed among the varying concentrations compared to lidocaine. The increase in fraction concentration showed an inverse relationship with the rapid movement and average speed, suggesting that excitation is a potential effector compound. As for social interaction, the decreased conspecific interaction time can be potentially explained by serotonin. Shoaling of zebrafish is an indicator of their health and well-being, decrease in interaction or observed poor social activity is associated with changes in brain function [62]. The neurochemistry of negative social interactions indicates a decrease in the serotonin and dopamine neurotransmitter levels as indicators, which is not observed to change locomotor activity [63, 64]. Suggestively, this neurochemical alteration may be induced by blocking VGSCs which inhibits presynaptic calcium channels, thereby halting the vesicular activity for the presynaptic release of neurotransmitters present in social activity [65].

The aggression phenotype in zebrafish involves reduced GABA signaling, serotonin deficits, an increase in fight-or-flight neurotransmitters (adrenaline, noradrenaline, and histamine), impaired dopamine signaling, steroids deficits, and activation of stress-related hormones [66]. Though from concurrent knowledge, deficits in serotonin are also associated with poor social interaction, which is also associated with VGSC blocking. Based on this concept, it contradicts the neurobehavior of the lidocaine-treated group. However, the absence of action potential in the neuromuscular junction [61] and the cholinergic effect of Nav blockers as presented by lidocaine are factors to consider for lack of aggression in the lidocaine and 200 ppm fraction-treated groups [54].

## Conclusion

The venom of the Philippine *Orphnaecus* tarantula species exhibited 11 distinct peaks, which were grouped into three major clusters: polar, semi-polar, and least polar fractions. The detected peaks may not be entirely concluded as proteins, as other substances can be detected across the 215-280 nm wavelength range. Nine fractions were subjected to neurobehavioral studies in zebrafish (*Danio rerio*) to determine the neuroactive fractions. Fraction 1 was further analyzed because it was previously reported to contain nonpeptidic compounds. Preliminary screening of all the fractions revealed that fraction four exhibited diazepam-like behavior with respect to its time spent on top, whereas Fractions 8 and 9 exhibited lidocaine-like behavior. The remaining venom fractions showed no significant behavioral difference with the negative control-NSS. Fractions 1, 4, and 8 were further evaluated by determining their respective dose-dependent responses, which followed the distinct effect of their corresponding positive controls. These findings highlight the potential of specific spider venom fractions to modulate distinct neurobehavioral phenotypes. Building on these initial results, future studies will focus on the purification and structural characterization of the active compounds, followed by functional validation using receptor-binding assays and electrophysiological techniques. Additional behavioral assays and structure-activity relationship (SAR) analyses will also be conducted to better understand the pharmacological profiles and therapeutic potential of these venom-derived molecules.

## Availability of data and materials

 The datasets generated and analyzed in this study are available upon reasonable request from the first author and corresponding author. Spider samples were deposited in Mindanao State University-Iligan Institute of Technology and Museum of Natural History of University of the Philippines Los Baños for further taxonomic study.
